# Seroprevalence of IgG Antibodies Against *Borrelia burgdorferi* Sensu Lato, *Anaplasma phagocytophilum*, and Tick-Borne Encephalitis (TBE) Virus in Horses in Southern Norway

**DOI:** 10.3390/microorganisms13040771

**Published:** 2025-03-28

**Authors:** Hanne Kloster, Camilla Stormo, Anita Haug Haaland, Snorre Stuen, Vivian Kjelland

**Affiliations:** 1 Department of Natural Sciences, Faculty of Engineering and Science, University of Agder, 4630 Kristiansand, Norway; hanne.kloster@uia.no (H.K.); camilla.stormo@uia.no (C.S.); 2 Department of Companion Animal Clinical Sciences, Faculty of Veterinary Medicine, Norwegian University of Life Sciences, 1433 Aas, Norway; anita.haug.haaland@mattilsynet.no; 3 Norwegian Food Safety Authority, Head Office, 0170 Oslo, Norway; 4 Department of Production Animal Clinical Sciences, Faculty of Veterinary Medicine, Norwegian University of Life Science, 4325 Sandnes, Norway; snorre.stuen@nmbu.no

**Keywords:** *Ixodes ricinus*, tick-borne pathogens, equine infection, immunoblot

## Abstract

*Ixodes ricinus* ticks play a crucial role as carriers of diseases, transmitting pathogens to vertebrate hosts, including horses. This study aimed to investigate the seroprevalence of IgG antibodies against *Borrelia burgdorferi* sensu lato (s. l.), *Anaplasma phagocytophilum*, and tick-borne encephalitis virus (TBE-virus) in equine sera collected in southern Norway. In total, sera from 331 horses stabled in four counties (Agder, Vestfold and Telemark, Vestland, and Viken) were analyzed by immunoblot. In total, 66% of the horses were IgG-seropositive for antibodies against one or multiple tick-borne pathogens. The highest seroprevalence was detected against *B. burgdorferi* s. l. (47%), followed by *A. phagocytophilum* (34%) and TBE-virus (10%). A significant difference between the counties regarding IgG antibodies against *A. phagocytophilum* was found, with the highest seroprevalence in horses stabled in the coastal areas of Agder and Vestland. In conclusion, the study demonstrates that horses in southern Norway are at high risk of contracting tick-borne infections.

## 1. Introduction

Ticks transmit a variety of pathogens affecting both human and animal health. Several tick species are present in Norway; however, *Ixodes ricinus* is the main tick vector of medical importance [1]. Norway constitutes part of the northern border for the geographical distribution of *I. ricinus* [2], and a recent expansion of *I. ricinus*’ range northward and to higher altitudes has been indicated [2,3,4]. However, this tick is still mainly found in the coastal regions from Viken County in the southeast to Brønnøysund in Nordland County in the north [2,3,4]. Further north and inland, the Scandinavian Mountains stand as defining features of the country’s landscape, and the cold temperatures and long-lasting snow cover play a pivotal role in preventing tick survival in these areas [5].

*I. ricinus* transmits an extensive range of viral, bacterial, and protozoan pathogens to vertebrate hosts [6]. To date, *Borrelia burgdorferi* sensu lato (s. l., subtypes: *B. afzelii*, *B. garinii*, *B. burgdorferi* sensu stricto, *B. valaisiana*, *B. finlandensis*, and *B. spielmanii*) [7,8,9], *Anaplasma phagocytophilum* [10], tick-borne encephalitis virus (TBE-virus, Western subtype) [11], *Neoerlichia mikurensis* [12], *Rickettsia helvetica* [13], *Borrelia miyamotoi* [14], and *Babesia* spp. [15] have been detected in host-seeking ticks collected in Norway. Furthermore, *Bartonella* spp. has been detected in ticks attached to moose [16]. However, *B. burgdorferi* s. l. and TBE-virus are responsible for the vast majority of human tick-borne infections in Norway [17], whereas *A. phagocytophilum* is the most reported cause of infection in domestic animals [18].

Knowledge regarding the occurrence and impact of tick-borne infections in horses is limited. Infections with *B. burgdorferi* s. l., *A. phagocytophilum*, or TBE-virus are, in most cases, found to be subclinical or present with general, diffuse, and usually self-limiting clinical signs [19,20,21]. However, infections with these pathogens can, in some cases, lead to more severe symptoms, such as acute febrile disease, equine neuroborreliosis, or encephalomyelitis [18,22,23,24]. Seroprevalence studies in domestic animals provide information on the risk of acquiring tick-borne infections in an area and increase awareness of tick-borne agents as potential causes of diffuse, general, or neurological clinical signs in horses. In addition, seroprevalence studies serve as an important surveillance tool from a One Health perspective.

The aim of this study was to investigate the seroprevalence of IgG antibodies against *B. burgdorferi* s. l., *A. phagocytophilum*, and TBE-virus in horses in southern Norway. Furthermore, the study aimed to evaluate the potential impact of factors that could increase the risk of acquiring tick-borne infection, such as geographic location and the horses’ age, sex, fur color, and paddock substrate.

## 2. Materials and Methods

### 2.1. Sample Collection

The study aimed to recruit 85 horses from each of the four counties (in January 2024, Vestfold and Telemark county was split into two counties, Vestfold and Telemark, whereas Viken county was split into three counties, Østfold, Akershus, and Buskerud) in southern Norway (Agder, Vestfold and Telemark, Vestland, and Viken) during the years 2021–2023, either through direct communication with the owners (Group 1) or through the Central Laboratory at the Norwegian University of Life Sciences (Group 2).

In Group 1, written consent for the inclusion and collection of blood samples for testing for IgG antibodies against tick-borne pathogens was obtained from the owners (Declaration of Consent S1). The inclusion criteria were as follows: geographic location of residence in one of the abovementioned counties and possible tick exposure (either in a paddock/outdoor area, when being trained, or when being led on walks). The owners were asked to complete a questionnaire regarding the horses’ age, color, sex, breed, home county, travel activity, instances of observed attached ticks on the horse, and any antibiotic treatment administered in association with tick bites (Declaration of Consent S1). The blood samples were collected by veterinarians and stored at room temperature for one to two hours to ensure clotting before centrifugation at 1800× *g* for 12 min. The serum was removed, aliquoted in microtubes, and stored at −20 °C until further analysis.

In Group 2, all the samples were residue sera from clinical samples donated by horse owners from horses residing in Viken County. The exact location of the stables was not available. Data such as age, breed, and sex were collected when available. Sera were stored at −20 °C until further analysis.

### 2.2. IgG-Antibody Testing

Sera were analyzed using the EUROLINE Tick-Borne Profile 1 Horse (IgG) kit (EUROIMMUN Medizinische Labordiagnostika AG, 23560 Lübeck, Germany), targeting IgG antibodies against *B. burgdorferi* s. l. (VIsE, OspC (p25) and p100), *A. phagocytophilum* (MSP-2), and TBE-virus (gpE), according to the manufacturer’s instructions. The immunoblot test is based on purified and biochemically characterized antigens applied to membrane strips. Each antigen is fixed to separate membrane fragments, and the antigen bands are located at defined positions on the membrane. Each strip has a control band that ensures the correct performance of the individual incubation steps. The results were automatically evaluated by the EUROLineScan program, and borderline results were interpreted as negative. According to the manufacturer, due to the high quality of the antigen substrate used, no cross-reaction of *B. burgdorferi* s. l. with other species is suspected. However, cross-reactions between *A. phagocytophilum* and *Anaplasma platys*, *Ehrlichia*, and *Rickettsia* have been reported. Furthermore, they state that cross-reactivity with other flaviviruses leading to false positive TBE-virus results cannot be excluded, but the use of recombinant antigens significantly reduces the probability of cross-reactions.

### 2.3. Statistics

Statistical analyses were performed using the Pearson Chi-square test and logistic regression analysis in STATA/SE 17.0 (STATA Corp., College Station, TX, USA, 2021); *p*-values < 0.05 were considered significant.

### 2.4. Spatial Visualization

The sample locations were mapped using R (version 2023.09.1+494) [25] with the ggplot2 (version 3.5.1) [26] and sf (version 1.0–20) [27] packages. Municipality boundaries were obtained from the rnaturalearth package, and sample locations were aggregated based on unique spatial coordinates. An inset map of Norway was included to indicate the zoomed-in region. The final map was exported as a high-resolution TIFF.

### 2.5. Ethical Approval

The study was ethically approved by the Norwegian Food Safety Authority, ID: FOTS id 27557.

## 3. Results

### 3.1. Demographic Characteristics

**Figure 1:**
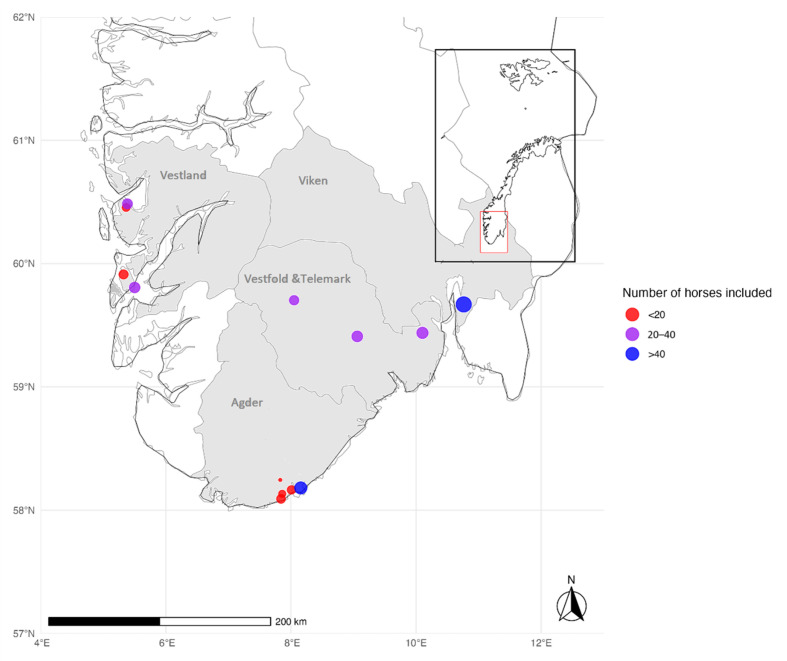
Blood samples were collected from horses residing in southern Norway: Agder, Vestfold and Telemark, Vestland, and Viken. The stables in Agder and Vestland were located within 15 km of the coast. In Vestfold and Telemark, the stables were located 60–120 km from the coast and defined as inland. The location of 71 of the 77 stables in Viken County (Group 2) was unknown, and coordinates for the Central Laboratory at the Norwegian University of Life Sciences were used as the location for Group 2. The number of samples at each location was represented using dot size, while categories (<20, 20–40, >40 samples) were color-coded in red, purple, and blue (the map was created in R Core Team (2023) and modified in Microsoft Paint).

The demographic characteristics of the horse population are summarized in Table S1. In total, 331 horses were included in the study: 85 horses residing in Agder, 85 in Vestfold and Telemark, 84 in Vestland, and 77 in Viken (Figure 1). The stables in Agder and Vestland were located < 15 km from the coast and defined as coastal, whereas the stables in Vestfold and Telemark were located 60–120 km from the coast and defined as inland. The location of the stables for 71/77 horses in Viken County was unknown. In total, 169 horses were from coastal areas and 91 from inland regions. All the horse owners in Agder, Vestfold and Telemark, and Vestland and 6 of the 77 horse owners from Viken were contacted through direct communication, and all the owners completed the questionnaire (Group 1, *n* = 260). The remaining samples from Viken were residual sera from clinical laboratory testing (Group 2, *n* = 71).

The age of the horses ranged from 4 months to 33 years (mean: 11.5 years, median: 11 years), with 136 females, 173 males (26 stallions and 147 geldings), and 22 of unreported sex. The horses were of a variety of breeds, with the Icelandic horse (*n* = 47), Coldblooded Trotter (*n* = 45), and Warmblooded Trotter (*n* = 41) being the most common (Table S2). The horses were stabled at equestrian centers (*n* = 150) or in privately owned stables (*n* = 62); however, information regarding stabling was not available for all horses (*n* = 119). The outside areas (paddocks) had grass (*n* = 94) or sand (*n* = 118) as substrate. In Group 1, the horse owners reported having removed ticks from 67% (57/85) of the horses stabled in Agder, 51% (43/85) in Vestfold and Telemark, and 45% (38/84) in Vestland. In addition, 4/6 owners in Viken had removed ticks from their horses. Only 1 of the 260 horse owners reported antibiotic treatment of the horse due to tick-borne infections. A total of 51% (132/260) of owners reported that their horse had not been outside their residential county, whereas 48% (124/260) reported travel activity outside their home county. Of these, 104 horses had been only in counties within southern Norway, whereas 20 had been abroad.

### 3.2. Seroprevalence of Tick-Borne Pathogens in Horses in Southern Norway

The seroprevalence of IgG antibodies against tick-borne pathogens in horses in southern Norway was high throughout the study area (Table 1). For each pathogen, the overall seroprevalence of *B. burgdorferi* s. l., *A. phagocytophilum*, and TBE-virus was 47%, 34%, and 10%, respectively.

**Figure 2:**
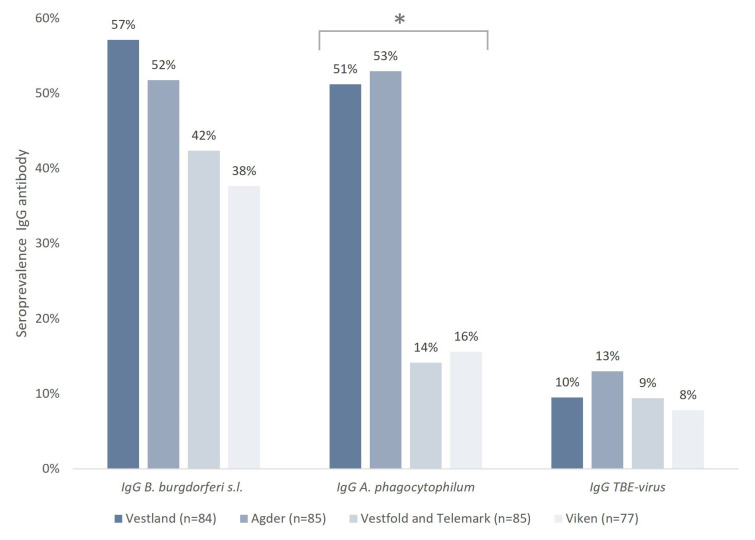
Seroprevalence of IgG antibodies against *B. burgdorferi* s.l., *A. phagocytophilum*, and TBE-virus in horses residing in southern Norway. * The differences between counties in the seroprevalence of *A. phagocytophilum* were statistically significant.

A significant difference in the seroprevalence of IgG *A. phagocytophilum* between counties was found (*p* = 0.005); the highest seroprevalence was observed in Agder (53%) and Vestland (51%), while the lowest was found in Viken (16%) and Vestfold and Telemark (14%) (Figure 2). However, no significant difference in the seroprevalence between counties was found for IgG *B. burgdorferi* s. l. (*p* = 0.054) or IgG TBE-virus (*p* = 0.731).

In total, 66% of the horses were IgG-seropositive for one or multiple tick-borne pathogens, ranging from 51% (Viken) to 80% (Vestland) (Table 2).

There was a significantly higher overall seroprevalence in horses stabled in coastal areas (51%, *n* = 169) than in those stabled in inland areas (26%, *n* = 85) (*p* = 0.006). Furthermore, a significantly higher seroprevalence of IgG antibodies against tick-borne pathogens was seen in horses using paddocks with grass substrate (82%, 77/94) than in horses using sand-covered paddocks (65%, 77/118) (*p* = 0.007). Moreover, IgG antibodies were identified in all age groups; however, seroprevalence was significantly higher in the 6–20-years age group (73%, 129/176) than in the <5-years (53%, 36/68) and >20-years (63%, 22/35) age groups (*p* = 0.041).

No significant differences were found between the most common breeds: the Icelandic horse (64%, 30/47), Coldblooded Trotter (51%, 23/45), and Warmblooded Trotter (66%, 27/41) (*p* > 0.05). Additionally, there were no significant differences regarding fur color (brown: 73% (79/109), red: 76% (29/38), white: 54% (37/20), black: 62% (16/26), beige: 80% (20/25), pinto/multicolored: 79% (11/14), *p* = 0.150) or sex (mare: 71%, 97/139, stallion: 64%, 13/26, gelding: 50%, 94/147, *p* = 0.081).

Blood samples were collected during winter (December–February), spring (March–May), or fall (September–November) (Table S1). When accounting for the county using logistic regression, no significant differences in seroprevalence related to the time of sampling were found.

## 4. Discussion

This study is, to our knowledge, the first to investigate the seroprevalence of IgG antibodies against three tick-borne pathogens in horses in Scandinavia. A high seroprevalence of IgG antibodies against *B. burgdorferi* s. l., *A. phagocytophilum*, and TBE-virus in horses was found throughout southern Norway. Here, *I. ricinus* population densities are generally higher in coastal areas than in inland areas [28]. Although the seroprevalence was significantly higher in horses stabled in coastal regions in the present study (<15 km from the coast; Vestland and Agder), a high seroprevalence was also found in horses stabled further inland (>60 km from the coast: Vestfold and Telemark), where a lower tick population density was found. However, despite the generally lower tick population density in inland regions, local *I. ricinus* populations exist, demonstrating the risk of equine infections also here. Furthermore, several owners reported traveling activity outside their resident county, and their horses may have been exposed to tick bites elsewhere. Nonetheless, in most cases, the traveling was within southern Norway, supporting the notion of a high risk of infection in this region.

The average equine seroprevalence of *A. phagocytophilum* in the present study was 34%. Previous studies from Norway and other Scandinavian countries have reported a lower seroprevalence in horses: 4% in Norway [29], 17% in Sweden [30], and 22% in Denmark [31]. Elsewhere in Europe, seroprevalence ranges from 0 to 73% [18,21,32,33,34,35]. In the present study, the seroprevalence of *A. phagocytophilum* was significantly higher in the counties of Agder and Vestland than in the counties of Vestfold and Telemark and Viken. The reason for this difference is unknown but may be due to variations in the prevalence of *A. phagocytophilum* in host-seeking ticks across the included regions, as previous studies have found significant variability (0–11.5%) [9,36,37,38,39]. It could also be due to the presence of different *A. phagocytophilum* variants [40], with varying pathogenicity and serological responses. Additionally, a speculative contributing factor to the high seroprevalence found in Agder and Vestland counties could be cross-reactivity with other agents present here. According to the manufacturer of the EUROLINE kit applied in the present study, cross-reactions with *A. platys*, *Ehrlichia*, and *Rickettsia* may occur. But, as *A. platys* is mainly distributed in the southern hemisphere [41], there are no reports of the detection of *Ehrlichia* species in Norway, and the prevalence of *Rickettsia* spp. in host-seeking ticks in Norway is low [9,13], we suggest that the most probable candidate for cross-reactions in this region is *N. mikurensis*. Although not demonstrated by the EUROLINE kit, cross-reactivity between *A. phagocytophilum* and *N. mikurensis* has been demonstrated when applying immunofluorescence assays (IFA) and polymerase chain reaction (PCR) [42]. A relatively high prevalence (up to 22%) of *N. mikurensis* has been detected in questing *I. ricinus* in coastal areas in southern Norway [9,12,36], which implies a risk of equine infection and possible cross-reactivity in serological analyses. Further research is necessary to investigate whether the high seroprevalence of *A. phagocytophilum* in equine sera in Agder and Vestland counties is due to cross-reactions. If it is true, the causative factors should be elucidated.

The average seroprevalence of antibodies against *B. burgdorferi* s. l. was 47%, which is at the high end of what has been found in horses elsewhere in Europe, where it ranges from 6 to 48% [18,32,33,35,43,44]. It is also higher than what has been reported in horses from other Scandinavian countries, with an average seroprevalence of 17% in Sweden [30] and 29% in Denmark [31]. Notably, the seroprevalence on the island Bornholm, an area previously found to have the highest tick density in Denmark, was 60% [31]. One explanation for the high seroprevalence in the present study may be a high tick population density combined with a high prevalence of *B. burgdorferi* s. l. in questing *I. ricinus*. The majority of the horses (65%) in Group 1 in the present study were stabled in tick-endemic areas, where previous studies have reported a high prevalence of *B. burgdorferi* s. l. in questing ticks (16–27%) [7,9,36]. This is also at the high end of what has been reported elsewhere in Europe; a meta-study found significant differences in the prevalence of *B. burgdorferi* s. l. in questing *I. ricinus* in different regions in Europe, with region averages ranging from 3 to 22% [45]. Although the high tick population density and the high prevalence of *B. burgdorferi* s. l. in ticks are probable causes for the high seropositivity found in the present study, further research is needed to verify these assumptions.

The seroprevalence of IgG antibodies against TBE-virus in horses in southern Norway was, on average, 10%. To our knowledge, this is the first Scandinavian study investigating the seroprevalence of TBE-virus in horses; however, elsewhere in Europe the seroprevalence is reported to range from 0 to 37% [46,47,48,49,50,51,52]. Previously, the seroprevalence of TBE-virus was investigated in cows from five farms across Norway, and antibodies were detected in 88% of the cows in the southernmost farm; however, no seropositivity was found elsewhere [53]. Furthermore, the seroprevalence in wild cervids has been reported to be 5% nationwide [54] and 41% in an endemic area [55], demonstrating the different risk levels in different regions within Norway. Accordingly, studies on human seroprevalence in Norway also vary from 0 to 2.4%, depending on the region [17,56]. Furthermore, although the average prevalence of TBE-virus in nymphal and adult *I. ricinus* in Norway is low (0.3% and 4.3%, respectively), it varies greatly between sites and counties (range of 0% to 21%) [57]. Hence, the risk of TBE infection shows considerable geographic variation in Norway, and human cases are reported in three of the four counties investigated in the present study (Agder, Vestfold and Telemark, and Viken) [58]. No report of human cases in Vestland County has been made so far, and a seroprevalence study among blood donors reported zero seropositivity in the county [59]. It is unknown why no human cases are reported from the southwestern coast of Norway, as ticks and TBE-virus are also prevalent here. Speculation includes underdiagnosis, the presence of less pathogenic virus variants, or differences in human behavior [57]. However, the present study found that the seropositivity in horses in Vestland is at the same level as in counties with human cases, demonstrating the risk of (equine) infection also in this region.

Although the seroprevalence of TBE-virus in horses found in the present study was within the range found in Europe, the choice of assay may have impacted the results. According to the manufacturer of the EUROLINE kit applied in the current study, cross-reactions with other flaviviruses leading to false positive TBV results cannot be excluded. Loping ill virus (LIV) is a flavivirus closely related to TBE-virus, and although there is a lack of detected infection in ticks [60] and rare reports of the disease [61], a seropositivity of 15% in cervids in a location in southern Norway was reported [57]. In a serological screening for TBE-virus in reindeer from the high-altitude areas of inland regions in Norway indicated that a flavivirus other than the TBE-virus, such as LIV, could be circulating among these animals [62]. In conclusion, although the use of recombinant antigens in this study significantly reduces the probability of serological cross-reactions between TBE-virus and LIV or other flaviviruses, this cannot be excluded. Further investigation is needed to verify the findings. Nevertheless, our study indicates that horses are at risk of acquiring viral infection after tick bites, and TBE should be included in the differential diagnosis list in horses with neurological symptoms.

Contrary to a previous study that found a higher seroprevalence in females than in geldings and stallions [63], the current study did not support this. Also, although Warmblooded and Coldblooded Trotters generally spend more time on racing tracks for competition training and less time in environments with potential tick exposure than breeds such as Icelandic horses, no correlation between breed and seropositivity was found, indicating that all breeds are at risk of tick infestation. Furthermore, across breeds, a significantly higher seroprevalence of IgG antibodies was found among horses with paddocks with a grassy substrate than among horses with paddocks with a sandy substrate, indicating that tick repellants should be considered for all breeds, especially if they have paddocks with a grassy substrate.

In the present study, only IgG antibodies were investigated. The detection of antibodies in sera does not necessarily mean that the animals have an acute infection. All the horses in Group 1 were assumed healthy and were asymptomatic for the pathogens considered here. Furthermore, all the horses in Group 2 had blood drawn for random diagnostic samples, and no suspicion of tick-borne disease in any of the horses was reported. Antibodies against tick-borne pathogens have previously been reported to last for several months to years in horses [63], which could contribute to the high seroprevalence reported in the present study. However, a high seroprevalence was found across all age groups, including foals, indicating a high risk of infection throughout southern Norway. At the same time, a high seroprevalence without confirmed clinical cases may indicate a low risk of disease after infection. Also, the overall high seroprevalence found in this study could relate to sampling bias. Possible tick exposure was an inclusion criterion for the horses recruited directly through their owners (Group 1); hence, these horses may have had a higher risk of tick bites than the general population.

The incidence rates of human tick-borne infections in Norway are increasing [58], demonstrating the importance of surveillance. Although horses may be used as sentinel animals, the findings should be considered carefully, as some horses have a travel history which may make the assessment of the risk of tick-borne infections in a certain area difficult. However, although less reliable than stationary sentinel animals, there are some advantages to using horses, including easy access to animals and good knowledge of their travel history.

## 5. Conclusions

The equine seroprevalence of antibodies against *B. burgdorferi* s. l., *A. phagocytophilum*, and TBE-virus were high throughout southern Norway. In contrast to the other pathogens, the seroprevalence of *A. phagocytophilum* showed marked geographical differences; however, further investigation is needed to determine whether this variation reflects differences in *A. phagocytophilum* prevalence in ticks in different areas or if it is due to the presence of different variants of *A. phagocytophilum* or cross-reacting agents in some regions. The study also found that paddock substrates influenced seroprevalence. Horses with grassy paddocks had a higher seroprevalence, indicating the need for tick repellents in such environments. No significant correlation was found between breed and seroprevalence or sex and seroprevalence, suggesting that all breeds and both sexes are at risk of tick infestation. In conclusion, the current study shows a high risk of acquiring tick-borne infections for horses stabled throughout southern Norway, indicating the need for increased awareness and the implementation of preventive measures to reduce tick infestation.

## Figures and Tables

**Table 1:** Seroprevalence of IgG antibodies against tick-borne pathogens in horses stabled in four counties in southern Norway.

	Total (N = 331) % (*n*)	Agder (N = 85) % (*n*)	Vestfold & Telemark (N = 85) % (*n*)	Vestland (N = 84) % (*n*)	Viken (N = 77) % (*n*)
*Bb*sl ^1^	47 (157)	52 (44)	42 (36)	57 (48)	38 (29)
*Ap* ^2^	34 (112)	53 (45)	14 (12)	51 (43)	16 (12)
TBEV ^3^	10 (33)	13 (11)	9 (8)	10 (8)	8 (6)

^1^ *Borrelia burgdorferi* sensu lato. ^2^ *Anaplasma phagocytophilum*. ^3^ Tick-borne encephalitis virus.

**Table 2:** Seroprevalence of IgG antibodies against tick-borne pathogens demonstrating single, double, or triple infection in horses.

	Total (N = 331)	Agder (N = 85)	Vestfold & Telemark (N = 85)	Vestland (N = 84)	Viken (N = 77)
	% (*n*)	% (*n*)	% (*n*)	% (*n*)	% (*n*)
*Bb*sl ^1^	26 (85)	19 (16)	31 (26)	24 (20)	30 (23)
*Ap* ^2^	13 (42)	20 (17)	5 (4)	18 (15)	8 (6)
TBEV ^3^	2 (8)	0	6 (5)	1 (1)	3 (2)
*Bb*sl + *Ap*	17 (55)	24 (20)	8 (7)	29 (24)	5 (4)
*Bb*sl + TBEV	4 (12)	6 (5)	2 (2)	4 (3)	3 (2)
*Ap* + TBEV	2 (8)	4 (3)	0	4 (3)	3 (2)
*Bb*sl + *Ap* + TBEV	2 (7)	6 (5)	1 (1)	1 (1)	0
Total positive	66 (217)	78 (66)	53 (45)	80 (67)	51 (39)

^1^ *Borrelia burgdorferi* sensu lato. ^2^ *Anaplasma phagocytophilum*. ^3^ Tick-borne encephalitis virus.

## Data Availability

The data that support the findings of this study are available on request from the corresponding author. The data are not publicly available due to privacy or ethical restrictions.

## Supplementary Materials

The following supporting information can be downloaded at: https://www.mdpi.com/article/10.3390/microorganisms13040771/s1, Declaration of Consent S1: Declaration of Consent, Table S1: Demographic characteristics from the questionnaire and information regarding the stables included, Table S2: An overview of horse breeds in the study.
