# Supplementary material for: Seroprevalence of IgG Antibodies Against Borrelia burgdorferi Sensu Lato, Anaplasma phagocytophilum, and Tick-Borne Encephalitis (TBE) Virus in Horses in Southern Norway
Source: Microorganisms. 2025 Mar 28;13(4):771. doi: 10.3390/microorganisms13040771 (PMC12029606; doi:10.3390/microorganisms13040771)
Supplement: Supplementary file 1 [file microorganisms-13-00771-s001.zip › Table S2 An overview of horse breeds in the study.pdf]

Table S2: An overview of horse breeds in the study

Horse breed	Total (N=331) (n)
American miniature	1
American Paint Horse	2
Arab horse,	4
Baroque pinto	1
Belgian sports pony	1
Coldblooded trotter	45
Connemara	7
Danish sports pony	4
Danish warmblood	11
Dole	11
Dutch sports pony	1
English fullblood	2
Fjording	16
Fredriksborger	1
German horse	2
German sports pony	1
Haflinger	1
Hanoverian horse	3
Holsteiner	9
Hungarian horse	1
Irish sports pony	3
Icelandic horse	47
Italian warmblood	1
Knabstrupper	2
Lithuanian warmblood	1
Lusitano	1
New Forest	1
Nordlandshest	9
Norwegian warmblood	8
Oldenburger	7
Pinto	1
Polish warmblood	1
Quarter horse	1
Shetlands pony	11
Sports pony	4
Swedish warmblood	1
Tinker horse	1
Trakehner	1
Warmblooded horse	9
Warmblooded trotter	41
Welsh cob Welsh Mountain	5
Westphalian	2
Zangersheid	2
Mixed breed	18
n/a*	28

*n/a: not available
