# Supplementary material for: Seroprevalence of IgG Antibodies Against Borrelia burgdorferi Sensu Lato, Anaplasma phagocytophilum, and Tick-Borne Encephalitis (TBE) Virus in Horses in Southern Norway
Source: Microorganisms. 2025 Mar 28;13(4):771. doi: 10.3390/microorganisms13040771 (PMC12029606; doi:10.3390/microorganisms13040771)
Supplement: Supplementary file 1 [file microorganisms-13-00771-s001.zip › Declaration of Consent S1.pdf]

S1: Declaration of Consent

I have received and understood the information about the project and have had the opportunity to ask questions regarding the project. I agree to participate in the project:

- ☐ Detection of antibodies against tick-borne pathogens in serum from horses
- ☐ Other relevant analyses

Information regarding my horse (voluntary):
Breed:
Age:
Colour:
Sex:

The horse is stabled in County: ....

Born in Norway:

- ☐ Yes
- ☐ No, born in (country):................................

Traveling activity outside the county:

- ☐ No
- ☐ Yes, which counties:.................................

Traveling activity outside the country:

- ☐ No
- ☐ Yes, which countries................................

Status of vaccination*:

- ☐ vaccinated
- ☐ unvaccinated

Have removed ticks from my horse:

- ☐ yes
- ☐ no

Antibiotic treatment due to tick-borne infection:

- ☐ yes
- ☐ no

I consider my horse health as:

- ☐ good
- ☐ moderate
- ☐ poor

Known diseases:
....
....

Signature:

*Vaccination regime in line with Norwegian recommendations
