# Supplementary material for: Seroprevalence of IgG Antibodies Against Borrelia burgdorferi Sensu Lato, Anaplasma phagocytophilum, and Tick-Borne Encephalitis (TBE) Virus in Horses in Southern Norway
Source: Microorganisms. 2025 Mar 28;13(4):771. doi: 10.3390/microorganisms13040771 (PMC12029606; doi:10.3390/microorganisms13040771)
Supplement: Supplementary file 1 [file microorganisms-13-00771-s001.zip › Table S1 Demographic characteristics from the questionnaire, and information rearding the stables included..pdf]

Table S1: Demographic characteristics from the questionnaire, and information regarding the stables included.

		Agder (N=85) % (n)	Telemark & Vestfold (N=85) % (n)	Vestland (N=84) % (n)	Viken (N=77) % (n)	Total (N=331) % (n)
**Sex**	Mare	48 (41)	38 (32)	31 (26)	48 (37)	41 (136)
	Stallion	1 (1)	6 (5)	8 (7)	17 (13)	8 (26)
	Gelding	46 (39)	57 (48)	58 (49)	14 (11)	44 (147)
	n/a*	5 (4)	-	2 (2)	21 (16)	7 (22)
**Age**	0-5 years	5 (4)	24 (20)	33 (25)	25 (19)	21 (68)
	6-10 years	24 (20)	19 (16)	10 (8)	21 (16)	18 (60)
	11-15 years	26 (22)	28 (24)	6 (5)	17 (13)	195 (64)
	16-20 years	22 (19)	12 (10)	5 (4)	25 (19)	16 (52)
	over 21 years	13 (11)	16 (14)	4 (3)	8 (6)	10 (34)
	n/a*	11 (9)	1 (1)	38 (32)	14 (11)	16 (53)
**Fur color**	Brown	38 (32)	44 (37)	43 (36)	5 (4)	33 (109)
	Red	15 (13)	20 (17)	10 (8)	-	12 (38)
	White/grey	18 (15)	13 (11)	13 (11)	-	1 (37)
	Black	6 (5)	7 (6)	17 (14)	1 (1)	8 (26)
	Beige	9 (8)	8 (7)	11 (9)	1 (1)	8 (25)
	Pinto/multicolored	5 (4)	7 (6)	5 (4)	-	4 (14)
	n/a*	9 (8)	-	4 (3)	92 (71)	25 (82)
**Breed***	Icelandic horse	12 (10)	18 (15)	11 (9)	17 (13)	14 (47)
	Coldblooded trotter	8 (7)	14 (12)	16 (13)	17 (13)	14 (45)
	Warmblooded trotter	2 (2)	15 (13)	17 (14)	17 (13)	12 (41)
	Danish Warmblood	9 (8)	4 (3)	-	-	3 (11)
	Fjording	7 (6)	2 (2)	11 (9)	-	5 (17)
	Dole	4 (3)	5 (4)	2 (2)	3 (2)	3 (11)
	Holsteiner	4 (3)	7 (6)	1 (1)	-	3 (10)
	Shetland pony	1 (1)	2 (2)	7 (6)	3 (2)	3 (11)
	Other	45 (38)	33 (28)	26 (22)	29 (22)	33 (110)
	n/a*	8 (7)	-	10 (8)	16 (12)	8 (27)
**Ticks observed on the horse**	Yes	67 (57)	51 (43)	45 (38)	4 (3)	43 (141)
	No	31 (26)	48 (41)	39 (33)	4 (3)	31 (103)
	n/a*	2 (2)	1 (1)	16 (13)	92 (71)	26 (87)
**Antibiotic treatment due to tick-borne infection**	Yes	-	1 (1)	-	-	0.3 (1)
	No	99 (84)	99 (84)	86 (72)	8 (6)	72 (246)
	n/a*	1 (1)	-	14 (12)	92 (71)	26 (85)
**Living in coastal areas (< 25 km from the coast)**	Yes	100 (85)	-	100 (84)	-	51 (169)
	No	-	100 (85)	-	-	26 (85)
	n/a*	-	-	-	100 (77)	23 (77)
**Stable**	Privately owned	12 (10)	35 (30)	26 (22)	-	19 (62)
	Equestrian center	79 (67)	25 (21)	74 (62)	-	45 (150)
	n/a*	9 (8)	40 (34)	-	100 (77)	36 (119)
**Outdoor area (paddock)**	Grass	49 (42)	35 (30)	26 (22)	-	28 (94)
	Sand	41 (35)	25 (21)	74 (62)	-	36 (118)
	n/a*	9 (8)	40 (34)	-	100 (77)	36 (119)
**Time of blood sampling**	Spring	22 (19)	60 (51)	-	-	25 (82)
	Summer	-	-	-	-	-
	Fall	42 (36)	14 (12)	-	91 (70)	44 (147)
	Winter	35 (30)	26 (22)	100 (84)	9 (7)	51 (170)

*n/a: not available
